# Forest Loss and the Biodiversity Threshold: An Evaluation Considering Species Habitat Requirements and the Use of Matrix Habitats

**DOI:** 10.1371/journal.pone.0082369

**Published:** 2013-12-04

**Authors:** Candelaria Estavillo, Renata Pardini, Pedro Luís Bernardo da Rocha

**Affiliations:** 1 Instituto de Biologia, Universidade Federal da Bahia, Salvador, Bahia, Brazil; 2 Departamento de Zoologia, Instituto de Biociências, Universidade de São Paulo, São Paulo, São Paulo, Brazil; University of California, Berkeley, United States of America

## Abstract

Habitat loss is the main driver of the current biodiversity crisis, a landscape-scale process that affects the survival of spatially-structured populations. Although it is well-established that species responses to habitat loss can be abrupt, the existence of a biodiversity threshold is still the cause of much controversy in the literature and would require that most species respond similarly to the loss of native vegetation. Here we test the existence of a biodiversity threshold, i.e. an abrupt decline in species richness, with habitat loss. We draw on a spatially-replicated dataset on Atlantic forest small mammals, consisting of 16 sampling sites divided between forests and matrix habitats in each of five 3600-ha landscapes (varying from 5% to 45% forest cover), and on an *a priori* classification of species into habitat requirement categories (forest specialists, habitat generalists and open-area specialists). Forest specialists declined abruptly below 30% of forest cover, and spillover to the matrix occurred only in more forested landscapes. Generalists responded positively to landscape heterogeneity, peaking at intermediary levels of forest cover. Open area specialists dominated the matrix and did not spillover to forests. As a result of these distinct responses, we observed a biodiversity threshold for the small mammal community below 30% forest cover, and a peak in species richness just above this threshold. Our results highlight that cross habitat spillover may be asymmetrical and contingent on landscape context, occurring mainly from forests to the matrix and only in more forested landscapes. Moreover, they indicate the potential for biodiversity thresholds in human-modified landscapes, and the importance of landscape heterogeneity to biodiversity. Since forest loss affected not only the conservation value of forest patches, but also the potential for biodiversity-mediated services in anthropogenic habitats, our work indicates the importance of proactive measures to avoid human-modified landscapes to cross this threshold.

## Introduction

 Loss of habitats, especially the conversion of tropical forests into agricultural and urban areas, is the main driver of the biodiversity crisis we are observing today [1]. Around 43% of the terrestrial world surface has been disturbed and the original vegetation converted into anthropogenic new habitats [2]. In tropical countries as Brazil, one third of the land has been converted and agricultural frontiers are still expanding [3]. Extinction rates in this century are estimated to be more than two orders of magnitude higher than background rates [4], and can soon be much higher if models of state shifts, which predict abrupt changes in ecological systems, are correct [2]. 

 While habitat loss is clearly a landscape scale process, affecting the survival and dispersal of spatially structured populations or metapopulations, most of our knowledge on its effects comes from studies conducted at the patch scale [5]. Advances in metapopulation [6] and landscape ecology [7,8] modeling suggest, however, that this approach is not adequate since responses at the patch scale are contingent on two main aspects of landscape context: the permeability of the newly-created anthropogenic habitats (i.e. the type and quality of the matrix surrounding habitat patches) [9], and the amount of remaining habitat at the landscape scale [7,10]. The influence of the amount of habitat at the landscape scale is linked to a series of non-linear relationships between this variable and habitat characteristics at the patch scale [5]. In simulated fragmented landscapes, the size of the largest patch decreases drastically below 60% of remaining habitat; the number of patches peaks at around 30% of remaining habitat; and the distance among patches increases exponentially around 10-20% of remaining habitat [5,7]

These structural landscape thresholds are expected to lead to species extinction thresholds, with species suddenly disappearing from all patches in a landscape. Both modeling [7,10–12] and empirical [10,13,14] studies suggest extinction thresholds are observed below 30% of remaining habitat, and thus would be linked to the exponential increase in the distance among patches at 10-20% of remaining habitat. However, the existence of a biodiversity or species richness threshold would require that most species in a community respond similarly to the loss of native vegetation, and despite some empirical evidence in favor of such a threshold [10,14], it is still the cause of much debate and controversy in the literature [e.g. [13,15,16].

 Much of this controversy comes from the fact that species in a community usually have different habitat requirements and thus are likely to respond differently to the loss of native vegetation and the expansion of anthropogenic habitats [17]. Indeed, species specific responses to habitat disturbances are the rule in patch scale studies comparing communities across distinct habitats [e.g. [18,19]. It is thus clear that evaluations of biodiversity thresholds caused by the loss of native vegetation should take into account both species habitat requirements and the response of species to the main anthropogenic habitats in the matrix. 

 As far as we are aware, there are no empirical studies that sampled simultaneously both the native vegetation and the main anthropogenic habitat in the matrix across a gradient of native vegetation loss at the landscape scale, considering species habitat requirements *a priori*. However, from the modeling studies mentioned previously we can predict at least three different responses to the loss of native forest at the landscape scale: (1) forest specialist species, which should not occur in the matrix, should go extinct below the threshold of 30% of forest cover given the exponential increase in the distance among patches at ~10-20% of remaining forest; (2) generalist species, which should occur both in forest and matrix, should be more common at around 30% of forest cover, where the number of patches is greatest and thus is landscape structural heterogeneity; and (3) open-area specialist should occur only in the matrix, and may be able to eventually invade depauperated forest patches in landscapes below the 30% threshold, where forest specialist should have gone extinct.

 In this paper we use a spatially replicated dataset (five 3600-ha landscapes ranging from 5% to 45% of remaining forest, each sampled in eight forest and eight matrix sites) and an *a priori* classification of species into habitat requirement categories (forest specialists, habitat generalists and open-area specialists) to evaluate these hypotheses on how species are expected to respond to the loss of native vegetation at the landscape scale. We chose as a model system the small mammal community of the Atlantic Forest. Small mammals, the most diverse group of mammals in the Neotropics [20], include species with different habitat requirements [21], and are good indicators of anthropogenic disturbances, displaying rapid and distinct responses to habitat fragmentation [10,18,22]. The Atlantic Forest, once one of the largest rainforests of the Americas, has been reduced to roughly 12% of its original cover [23], and nowadays is considered to be one of the five most critical hotspots of biodiversity on a global scale [24]. 

We thus aim at helping to advance our knowledge on the existence of a biodiversity threshold, which should depend on how many species in a community belongs to each of the groups with distinct habitat requirements and distinct responses to the loss of native vegetation. Our results suggest that despite differential responses to habitat loss among forest specialist, habitat generalist and open-area specialist species, a strong biodiversity threshold can be observed across fragmented landscapes.

## Methods

 This study is part of a larger multi-taxa project carried out by a group of researchers from the Universidade Federal da Bahia - UFBA, which focus on the effects of habitat loss on eight distinct taxonomic groups in the Atlantic Forest of Bahia, Brazil. 

### Ethics statement

Trapping, handling and specimens collection were approved by IBAMA - Instituto Brasileiro do Meio Ambiente e dos Recursos Naturais Renováveis (license number 12023-3). Because our study involved capture and handling of small mammals in the field, it did not receive an approval in advance from the Committee for Animal Use of the Institute of Biology -UFBA (Comissão de Ética no Uso de Animais – CEUA, http://www.ceua.ufba.br), which only requires approval for studies on vertebrates that include experimentation (e.g. maintenance in captivity, injection of drugs, or surgery). However, retrospective approval was subsequently obtained from the UFBA CEUA (approval number 13/2013). Trapping, handling and euthanasia methods followed the guidelines of the American Society of Mammalogists Animal Care and Use Committee. No in vivo procedures were carried out prior to humane euthanasia of animals executed by gradual hypoxia with CO2. The specimens collected are deposited at the Museum of Zoology, UFBA, in compliance with national laws (IN 154/IBAMA). We did not sample either protected areas or species.

### Study areas and sampling design

**Figure 1:**
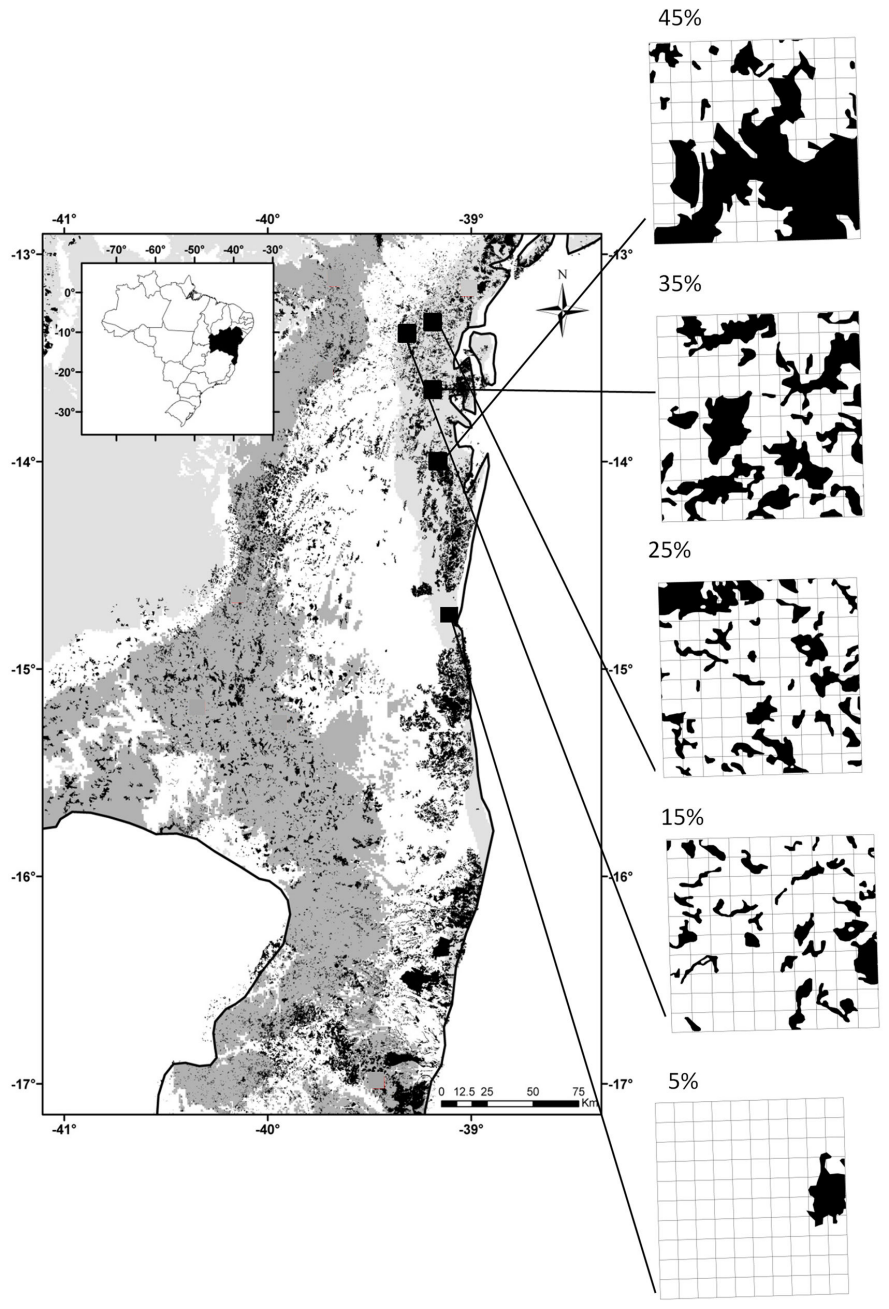
Distribution of Atlantic forest remnants in Southern Bahia and in the five studied landscapes. Black – forest remnants; dark gray – area proposed as historical forest refugia; light gray – non-refugial area; squares – location of the five studied landscapes.

 The portion of Atlantic Forest in the state of Bahia (Brazil) is considered to be one of the five centers of regional endemism in the biome [25]. It is currently dominated by secondary forest surrounded by a matrix of pasture intermixed with a variety of tree crops, including cocoa, rubber, bananas, palm oil, eucalyptus and coffee, mainly in privately-owned land [26]. Our study region comprises the coastal strip of Atlantic Forest south to Todos os Santos Bay (N-S 13°00' -14°50' and E-W 39°00' -39°30') (Figure 1). This region has a common biogeographic history: it is part of the northern portion of the Atlantic Forest, which has a different history from the southern portion [27], and it is restricted to the coast, supposed to be a non-refugial area during the Quaternary [28]. 

 From the study region, we sampled five landscapes of 6 x 6 km (3600 ha) varying in the amount of remaining forest (5%, 15%, 25%, 35% and 45%), in all of which altitude was less than 300 m, forest remnants were in mid to advanced stages of regeneration and the matrix was dominated by open areas. The study region was divided in a grid of 6 x 6 km landscapes. For all these landscapes, and larger surrounding areas of 18 x 18 km, we calculated forest cover, the size of the largest forest patch (Largest Patch Index; [29]), and the percentage of the matrix that is non-forested and non-urban based on recent satellite images (2005-2008) from the “Atlas dos Remanescentes Florestais da Mata Atlântica” (www.sosma.org.br and www.inpe.br). We controlled both forest cover and the size of the largest forest patch in the surrounding, larger areas, since both could act as source areas, thus considering only the 6 x 6 km landscapes where forest cover and the size of the largest forest patches were larger than the observed in the surroundings (18 x 18 km). We also controlled the permeability of the matrix by considering only landscapes where at least 80% of the matrix presented low permeability, with vegetation height less than 2 m (thus not computing urban areas, tree plantations, such as cacao, pines, eucalyptus, and rubber trees, and young secondary forests). We then chose the sampled landscapes by randomizing their spatial distribution to avoid correlation between geographical position and percentage of forest cover (Figure 1).

 The five selected landscapes (Figure 1) were located in the following municipalities: Ilhéus (IOS, 5% forest cover), Presidente Tancredo Neves (PTN, 15%), Valença (VAL, 25%), Nilo Peçanha (NLP, 35%) and Camamú (CAM, 45%). Each of the five landscapes was divided into grids of 100 plots of 600 x 600 m classified as either forest or matrix plots. We then randomly chose 8 plots of each type (16 per landscape) to locate our sampling sites, which were checked in the field to guarantee that forests corresponded to secondary forest in mid to advanced stages of regeneration and the matrix to non-urban, open areas with vegetation height less than 2 m. We also ensured there was a minimum distance of 30 m from all sampling sites to forest edge. 

Our sampling is spatially rather than temporally replicated, and was designed to provide a comparable, standardized snap-shot of the small mammal communities across landscapes with different amounts of forest cover. However, we controlled for temporal variation by sampling all landscapes within the same season and year, and all sites within each landscape simultaneously (see below).

### Data collection

 We sampled small non-volant mammals (rodents and marsupials) in all 80 sites (8 forest and 8 matrix sites in each of the five landscapes) using both pitfall traps (35 l) and medium-sized live traps similar to Sherman (10 x 10 x 30 cm) and Tomahawk (15 x 17 x 45 cm). In each sampling site we established two 100-m long trap lines, 5 m apart from each other: one containing 10 equidistant pitfall traps connected by a 50-cm high plastic drift fence, and the other containing 20 equidistant live traps on the ground (10 of each type). We baited the traps with a mixture of peanut butter, sunflowers seeds, oat grains, palm-oil and sardines. We conducted one capture session of eight days in each site, totaling 240 traps x night per site, 3840 traps x night per landscape and 19200 traps x night in the whole study. All sites and landscapes were sampled in the dry season, between January to March and September to November of 2011. The sixteen sites in a landscape were sampled simultaneously and thus the sampling of each landscape took around 10 days. 

 Captured specimens were collected and are deposited at the Museum of Zoology of UFBA. We identified small mammals to species level following the literature and consulting the specialists Yuri Leite and Leonora Costa from Universidade Federal do Espirito Santo. 

### Data analysis

 Captured species were *a priori* classified into three categories of habitat requirements – forest specialists, habitat generalists, and open-area specialists - based on previous information available in the literature on habitat use and on geographical distribution (see Text S1 for a detailed definition of each habitat requirement category and the list of references for classifying each species, and Table S1 for the list of species in each habitat requirement category). 

 For each of the three habitat requirement categories as well as for the entire small mammal community, we quantified: (1) the number of species (alpha diversity) and the total number of captured individuals (abundance) in each forest and matrix site, and (2) the total number of captured species (gamma diversity) within the set of eight forest sites, the set of eight matrix sites, and the set of all 16 sites in each landscape (irrespective of habitat type). We then plotted across landscapes with increasing amount of remaining forest (5% to 45%): (1) the mean and the 95% confidence interval of the abundance and alpha diversity among sites in forests, in matrix habitats, and in the landscape (irrespective of habitat type); (2) the gamma diversity per each habitat type and per landscape.

To highlight differences in species composition between habitats and landscapes, we also run a Non-metric Multidimensional Scaling (NMS, two axes), using the Jaccard similarity index on the presence/ absence matrix of all small mammal species per habitat type and landscape.

## Results

 A total of 242 individuals of 24 different species were captured; 91 individuals representing eight forest specialist species, 66 individuals representing eight habitat generalist species, and 85 individuals representing eight open-area specialist species. 

**Figure 2:**
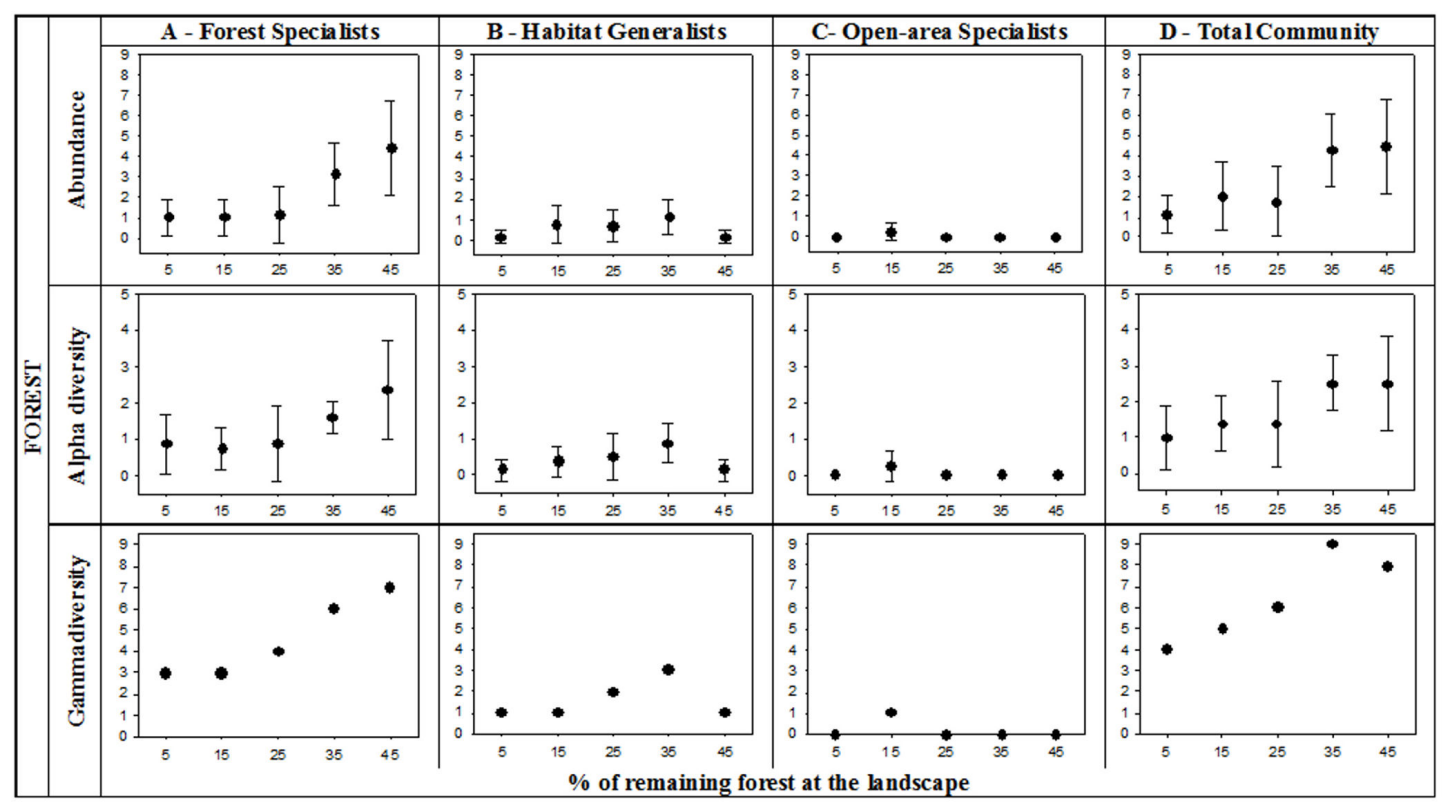
Abundance, alpha diversity and gamma diversity in forests across landscapes with increasing amount of remaining forest. For abundance and alpha diversity, the mean and 95% confidence interval among the eight surveyed sites per landscape are shown. A. Forest specialist species; B. Habitat generalist species; C. Open-area specialist species; D. Small mammal community.

In forests, forest specialist species were the dominant assemblage, with the highest abundance, alpha diversity and gamma diversity, followed by habitat generalist species (Figure 2). Open-area specialist species were not found in forests, except from one individual in the 15% forest cover landscape (Figure 2C). The abundance, alpha diversity and gamma diversity of forest specialist species declined similarly as a function of forest loss at the landscape scale, dropping from the 45 to the 25% forest cover landscape, and reaching a low plateau in the landscapes with 25 to 5% forest cover (Figure 2A). Abundance was consistently lower in the 5, 15, and 25% compared to the 45% forest cover landscape (no overlap in the confidence intervals), and gamma diversity was less than half in the 5 and 15% compared to the 45% forest cover landscape (Figure 2A). Habitat generalist species, on the other hand, presented a different pattern in forests: abundance, alpha diversity and gamma diversity were lowest at both extremes of the forest cover gradient (5 and 45%), and increased slightly from 15 to 35% forest cover (Figure 2B). As a result of the combination of these two distinct patterns, the abundance and alpha diversity of the whole small mammal community in forests were slightly lower in the least (5-25% forest cover) compared to the most (35 and 45%) forested landscapes, and gamma diversity peaked at the 35% forest cover landscape (Figure 2D).

**Figure 3:**
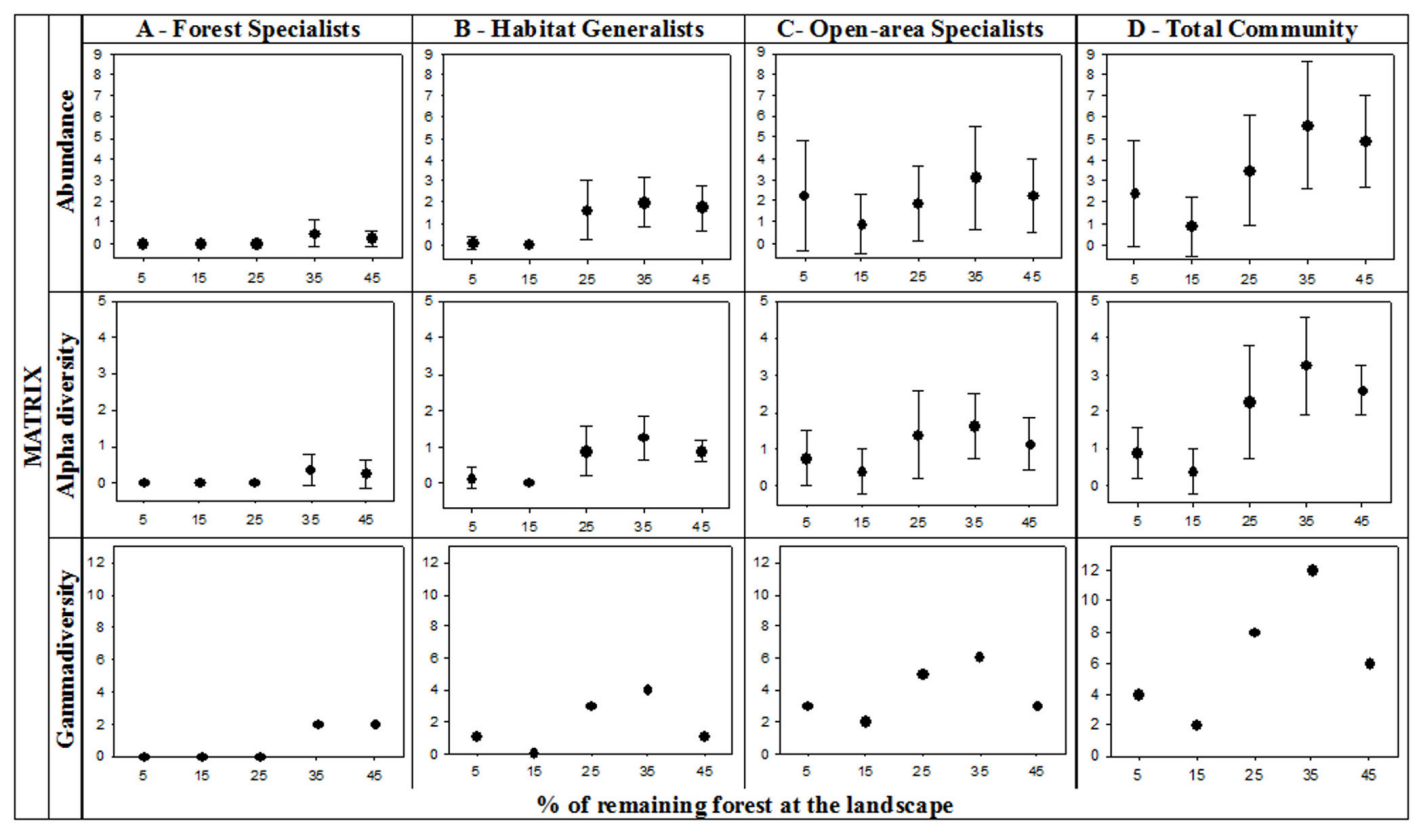
Abundance, alpha diversity and gamma diversity in the matrix across landscapes with increasing amount of remaining forest. For abundance and alpha diversity, the mean and 95% confidence interval among the eight surveyed sites per landscape are shown. A. Forest specialist species; B. Habitat generalist species; C. Open-area specialist species; D. Small mammal community.

In the matrix, the dominant assemblage was the open-area specialist species, followed by the habitat generalist species (more common in the matrix than in forests), with the forest specialist species only rarely captured (Figure 3). In fact, only a few individuals and species of forest specialists occupied the matrix and only in the two most forested landscapes (35 and 45% forest cover) (Figure 3A); hence, forest specialists were present in the matrix only in the landscapes where they were abundant and diverse in forests (Figure 2A). Habitat generalists were more common in the matrix than forest specialists. However, they also occupied the matrix mainly in the three (instead of the two as for forest specialists) most forested landscapes (25, 35 and 45% forest cover landscape, Figure 3B). Their gamma diversity in the matrix, however, also peaked in the intermediate forest cover landscapes as in forests (25 and 35 % forest cover landscapes, Figure 2B, Figure 3B). Open-area specialist species, in contrast, were present in the matrix in all landscapes, irrespective of forest cover (Figure 3C). Their abundance and alpha diversity, however, varied widely within landscapes (size of confidence intervals in Figure 3C) and did not vary consistently across the gradient of forest loss (Figure 3C). Similarly to the observed for the habitat generalists, their gamma diversity in the matrix peaked in the intermediate forest cover landscapes (25 and 35 % forest cover landscapes, Figure 3C). As a result of the combination of these distinct responses, abundance, alpha diversity and gamma diversity of the whole small mammal community in the matrix were similar to the observed in forests: the three variables were generally lower in the two (instead of the three as in forests) least forested landscapes (5 and 15%) compared to the three most (25, 35 and 45%) forested landscapes, and the three variables (instead of only gamma diversity as in forest) peaked at the 35% forest cover landscape (Figure 3D).

**Figure 4:**
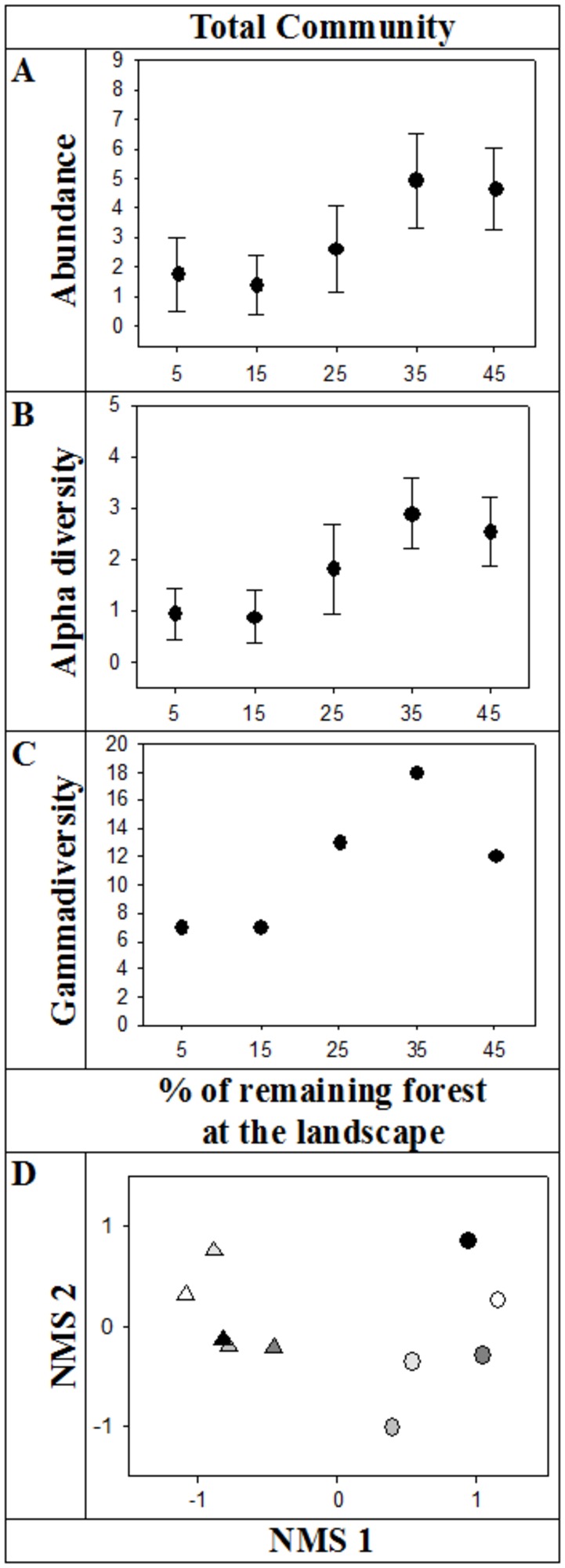
Abundance, diversity and composition of the small mammal community across landscapes with increasing amount of remaining forest. A. Abundance (mean and 95% confidence interval). B. Alpha diversity (mean and 95% confidence interval). C. Gamma diversity. D. Biplot of 2D NMS ordination of the presence/ absence matrix of all small mammal species in each habitat and landscape. Dot – forest; triangle – matrix; from white to black – increasing amount of remaining forest at the landscape.

It is important to notice though that, despite this similarity in abundance and diversity of the small mammal community between forests and the matrix across the gradient of forest loss, the differential response of the three habitat requirement categories led to very distinct small mammal communities in these two habitats (Figure 4D). In forests, the high abundance and diversity in the most forested landscapes are mainly due to forest specialists and secondarily due to habitat generalists (Figure 2), while it is mainly due to open-area specialists and habitat generalists in the matrix (Figure 3).

Finally, considering all three habitat requirement categories and both habitat types together, the small mammal community presented a clear threshold across the gradient of forest loss, with both abundance and alpha diversity being consistently higher in the two most forested (35 and 45%) compared to the two less forested (5 and 15%) landscapes, with intermediate values in the 25 % forest cover landscape (Figure 4A, Figure 4B). The pattern for gamma diversity was similar, but with a clear peak at the 35% forest cover landscape (Figure 4C). 

## Discussion

Our work used a spatially-replicated dataset to investigate the differential responses of groups of species with different habitat requirements to both matrix habitats and forest loss, allowing us to investigate the existence of a biodiversity threshold. Below, we first discuss the differential responses to forest loss among small mammal assemblages with different habitat requirements (forest specialists, habitat generalists and open-area specialists). We then discuss the observed species spillover between forests and anthropogenic habitats, and the observed different ecological thresholds across the gradient of habitat loss. Lastly, we point out our main conclusions and the potential consequences of the biodiversity threshold we observed. 

### Differential responses of habitat requirement categories to forest loss

As expected, forest specialist species declined abruptly in forests of landscapes below 30% of remaining forest, a similar threshold to that observed in another small mammal empirical study in the Atlantic forest [10,13]. These results are also comparable to observed trends for other taxonomic groups [14,30], and corroborate the idea that species-specific extinction thresholds are similar among habitat specialist species and are associated with the exponential increase in the distance among forest patches around ~20% of remaining habitat [8], which would prevent dispersal among sub-populations [31].

Forest specialists were much more common in forests than in anthropogenic habitats, as expected. They occurred in the matrix, but only in forested landscapes above the 30% forest cover threshold, i.e. in landscapes where they are common and abundant in forests. This finding corroborates the importance of “cross habitat spillover” in fragmented landscapes [32], and indicates that for forest specialists this process depends on landscape forest cover and thus on the overall size of source populations. 

Habitat generalist species, in contrast, apparently responded to another structural landscape threshold - the increased landscape structural heterogeneity - associated with the peak in the number of forest patches in landscapes at around 30% of remaining habitat [5,11]. This finding has been previously hypothesized in theoretical [33,34] and simulation studies [35], but has rarely been tested in empirical studies [36,37]. In contrast to specialist species mainly affected by habitat amount, generalists thus seem to be affected mainly by habitat and landscape heterogeneity [38]. 

It is important to mention, though, that the pattern of increased abundance and diversity of generalists in intermediately-forested landscapes was most evident in forests, while in the matrix habitat generalists were most common and diverse in more forested landscapes, as forest specialists. Therefore, even considering habitat generalists, the matrix of highly deforested landscapes supports very few species, with potential consequences for ecological functioning in the anthropogenic habitats of these degraded landscapes (see below). 

Open-area specialist species, on the other hand, were the dominant assemblage in the matrix, as expected. Interestingly, they did not spillover to forests, irrespective of landscape forest cover, indicating a resistance of forests to invasion [39] even in highly deforested landscapes where forest patches harbor extremely poor communities. Moreover, their gamma diversity was highest in intermediately-forested, rather than highly deforested landscapes, indicating that open-area specialists, although not invading forest patches, may benefit from landscape structural heterogeneity. Finally, their abundance and alpha diversity did not increase with forest loss, meaning that an expansion of open areas did not lead to the propagation of the populations of these species as it has been previously hypothesized [40]. However, it is important to notice that we did not sample highly forested landscapes, where open-area specialist species may not be able to persist. Only in these highly forested landscapes, where the matrix comprises less than 30% of the landscape (i.e. > 70% forest cover landscapes), matrix patches will be very small and isolated. Future studies on the effects of forest loss should extend the amplitude of the forest cover gradient under analysis to test if open-area specialist species are not able to persist in highly forested landscapes, where open-area patches are very small and isolated. 

### Cross habitat species spillover across a gradient of forest loss

From the distinct responses among habitat requirement categories described above, interesting patterns of species spillover between habitats in fragmented landscapes emerge. We observed that cross habitat species spillover occurred only from forests to the matrix (and not from the matrix to forests), but just in forested landscapes, with potential consequences to ecological functioning and services in anthropogenic habitats. 

Firstly, benefits from ecosystem services associated with the presence of forest specialist species in anthropogenic habitats should depend on forest cover, occurring only in forested landscapes. In the case of small mammals, for instance, the cross habitat spillover of forest specialist species may result in an important ecosystem service - disease regulation. The main reservoirs of hantavirus causing the fatal Hantavirus pulmonary syndrome in humans are usually generalist rodents [41], and there is evidence for a dilution effect propitiated by the diversity of small mammal communities: the presence of less efficient hosts leads to a lower prevalence of the virus in the main host and a lower risk of disease transmission [42]. 

Secondly, there is no evidence in this study of ecological compensation or competitive release in either forests or the matrix of highly deforested landscapes, both harboring poor (less rich and abundant) communities. Again this indicates the potential for losing biodiversity-mediated ecosystem services in highly deforested landscapes, as for example the regulation of pest outbreaks, which are commonly observed in certain types of crops and rodents in South America and other parts of the world [43]. 

### Ecological thresholds across a gradient of forest loss

Our work extends previous findings on the existence of extinction thresholds in forests across fragmented landscapes [10,13,14]. We showed that a biodiversity threshold, below 30% of habitat, occurs at the landscape scale and in both forests and the matrix, irrespective of differences in responses to forest loss among habitat requirement categories. Although species responded in a variety of ways, not only forest specialists were negatively affected by forest loss, but also habitat generalists (especially inside forests). Moreover, open-area specialists did not proliferate or spillover to forests in highly deforested landscapes. The drastic drop in biodiversity in the entire landscape as well as in forests was observed below 30% of forest cover, as suggested by landscape ecology theory [7]. The threshold in the matrix, however, was observed latter across the gradient of forest loss, around 20% of forest cover. 

 This study also suggests that total gamma diversity peaks just above the biodiversity threshold due to habitat generalist and open-area specialist species. Although this increase has been hypothesized in the literature [33], as far as we know it has not been tested or corroborated in empirical studies. This finding strongly suggests the importance of landscape heterogeneity to biodiversity [44]. 

 It is important to note, however, that the spatial scale at which such thresholds are observed should depend on the group of organisms under consideration. Since the spatial scale at which organisms are affected by landscape structure depends on their vagility and dispersal ability [45,46], for organisms larger than small mammals the thresholds may be apparent only when considering landscapes larger than the 3600-ha landscapes we studied.

### Conclusions and implications

Our work corroborates previous findings on the importance of niche breadth and habitat requirements as determinants of ecological responses to the loss of native vegetation [10,18,47] and of extinction risk [48,49]. It highlights that the cross habitat species spillover between forests and anthropogenic habitats may be asymmetrical and contingent to landscape context, occurring mainly from forests to the matrix and only in forested landscapes. More importantly, our landscape-scale sampling design incorporating both forest and the matrix, as well as the consideration of habitat requirement categories, allowed us to demonstrate the potential for biodiversity thresholds in human-modified, fragmented landscapes, and to bring evidence of the importance of landscape heterogeneity to biodiversity.

Both the asymmetrical, context dependent cross habitat spillover, and the associated biodiversity threshold suggest that forest loss strongly affects the conservation value of forest patches as well as the potential for biodiversity-mediated services, such as disease regulation and pest control, in anthropogenic habitats. They thus indicate the importance of proactive measures to avoid that human-modified landscapes cross this threshold and the limitation of reactive measures, such as ecological restoration, in highly deforested landscapes [10].

## Supporting Information

Text S1 ***Apriori* classification of small mammal species into habitat requirement categories.** Detailed definition of each habitat requirement category, and the list of references for classifying each species.
(DOC)

Table S1 **List of small mammal species in each habitat requirement category.** List of captured small mammal species, showing their classification for each of the two criteria (habitat use and geographical distribution) and the final habitat requirement classification.
(DOCX)
