# Supplementary material for: Forest Loss and the Biodiversity Threshold: An Evaluation Considering Species Habitat Requirements and the Use of Matrix Habitats
Source: PLoS One. 2013 Dec 4;8(12):e82369. doi: 10.1371/journal.pone.0082369 (PMC3853156; doi:10.1371/journal.pone.0082369)
Supplement: Text S1 — Apriori classification of small mammal species into habitat requirement categories. Detailed definition of each habitat requirement category, and the list of references for classifying each species. (DOC) [file pone.0082369.s001.doc]

**Text S1*. A priori* classification of small mammal species into habitat requirement categories.** Detailed definition of each habitat requirement category, and the list of references for classifying each species.

We used two criteria to classify *a priori* each captured species into three habitat requirement categories:

(1) *Habitat use* - data in the literature on species occupancy and/or abundance across different habitats (including native forests, and open, anthropogenic or altered habitats). This information was mainly based on Rossi (2011), which is a comprehensive and recent compilation of all published articles and Master or PhD thesis on the ecology of several Atlantic Forest small mammals. For captured species not reviewed by Rossi (2011) we used additional published articles:

*Hylaeamys laticeps* (Pardini 2004; Weskler et al 2006)

*Trinomys setosus* (Attias et al 2009)

*Marmosa murina* (Pardini 2004, Bezerra et al 2009)

*Micoureus demerarae* (Costa 2003, Pardini 2004)

*Monodelphis domestica* (Fernandes and Pires 2006)

*Cavia aperea* (Voss et al 2001)

*Pseudoryzomys simplex* (Voss and Myers 1991)

*Cerradomys vivoi* (Percequillo et al 2008)

(2) *Geographical distribution* – compilations in the literature on localities of collected specimens or distribution across biomes in Brazil, from which we evaluated if species range is centered in either the forested biomes (the Amazon and the Atlantic Forest) or the open, savanna-like biomes that separate the two forested biomes (the Caatinga and the Cerrado), or clearly encompasses both forested and open biomes. Species with geographical range centered in either forested or open biomes may also occur in the transitional areas between the two types of biomes. This procedure is a modification of that used by Pardini et al. (2009, 2010). This geographical information was obtained from Paglia et al. (2012), a recent annotated check list of Brazilian mammals, and from Carmignotto (2004), a comprehensive compilation of the distribution, biogeography and faunistic affinities of small mammals in the main open, savanna-like biome (the Cerrado) that separates the two forested biomes in Brazil.

Using these two criteria, habitat requirement categories were defined as:

Forest specialists - species that have occupancy and/or abundance restricted to, or higher in, forests than in open, anthropogenic or altered habitats, and at the same time have their geographical range centered mainly on forested biomes, the Amazon and the Atlantic forest (not occurring in the open, savanna-like biomes that separate the two forested biomes, or if occurring, just in transitional areas between forested and open biomes).

Habitat generalists - species that have occupancy and/or abundance similar between forests and open, anthropogenic or altered habitats, and have their geographical range either encompassing both forested and open-savanna-like biomes or centered mainly on forested biomes.

Open-area specialists - species that have occupancy and/or abundance restricted to, or higher in, open, anthropogenic or altered habitats than in forests, and have their geographical range either centered mainly in the open, savanna-like biomes that separate the two forested biomes or encompassing both forested and these open biomes, or are exotic, introduced species.

The habitat requirement classification for each species can be found in Table S1.

**References**

Attias N, Raíces D, Pessoa F, Albuquerque H, Jordão-Nogueira T, Modesto T and Bergallo H (2009). Potential distribution and new records of *Trinomys* species (Rodentia:Echimyidae) in the state of Rio de Janeiro. Zoologia (Curitiba, Impr.) 26(2): 305-315.

Bezerra AMR, Carmignotto AP and Rodrigues FHG (2009). Small Non-Volant Mammals of an Ecotone Region between the Cerrado Hotspot and the Amazonian Rainforest, with Comments on Their Taxonomy and Distribution. Zoological Studies 48(6): 861-874

Carmignotto AP (2004) Pequenos mamíferos terrestres do bioma Cerrado: padrões faunísticos locais e regionais. PhD Thesis, University of São Paulo, São Paulo.

Costa LP (2003) The historical bridge between the Amazon and the Atlantic Forest of Brazil: a study of molecular phylogeography with small mammals. J Biogeogr 30: 71–86. doi: 10.1046/j.1365-2699.2003.00792.x

Fernandes F and Pires, AS. (2006) Perspectivas para a sobrevivência dos marsupiais brasileiros em fragmentos florestais: o que sabemos, e o que ainda precisamos aprender? In: Os Marsupiais do Brasil: biologia, ecologia e evolução. Cáceres NC and Monteiro-Filho ELA Eds.

Paglia A, Fonseca G, Rylands A, Herrmann G, Aguiar L, et al. (2012) Lista Anotada dos Mamiferos do Brasil/ Annotated Checklist of Brazilian Mammals. 2ª Edição / 2nd Edition. Occasional Papers in Conservation Biology, No. 6. Conservation International, Arlington, VA. 76pp

Pardini R, Bueno AdA, Gardner TA, Prado PI, Metzger JP. (2010) Beyond the Fragmentation Threshold Hypothesis: Regime Shifts in Biodiversity Across Fragmented Landscapes. PLoS One 5(10): e13666. doi:10.1371/journal.pone.0013666

Pardini R (2004). Effects of forest fragmentation on small mammals in an Atlantic forest landscape. Biodivers Conserv 13: 2567-2586.

Pardini R, Faria D, Accacio GM, Laps RL, Marianno-Neto E, et al. (2009) The challenge of maintaining biodiversity in the Atlantic forest: a multi-taxa conservation assessment of specialist and generalist species in an agro-forestry mosaic in southern Bahia. Biol Conserv 142: 1178–1190. doi: [10.1016/j.biocon.2009.02.010](http://dx.doi.org/10.1016/j.biocon.2009.02.010)

Percequillo A, Hingst-Zaher E and Bonvicino C. (2008). Systematic Review of Genus Cerradomys Weksler, Percequillo and Voss, 2006 (Rodentia: Cricetidae: Sigmodontinae: Oryzomyini), with Description of Two New Species from Eastern Brazil. Am Mus Novit N°3622. 46p.

Pires AS, Lira PK, Fernandez FAS, Schittini GM, Oliveira LC. (2002) Frequency of movements of small mammals among Atlantic forest fragments in Brazil. Biol Cons 108: 229–237

Rossi NF (2011). Pequenos mamíferos não-voadores do Planalto Atlântico de São Paulo: Identificação, história natural e ameaças. 400p. Master Dissertation. Instituto de Biociências da Universidade de São Paulo.

Voss RS, Lunde DP and Simmons NB (2001) The Mammals of Paracou, French Guiana: A Neotropical Rainforest Fauna. Part I: Nonvolant Species. Bull Am Mus Nat Hist 263: 1-236.

Voss RS and Myers P (1991) *Pseudoryzomys simplex* (Rodentia: Muridae) and the significance of Lund´s collections from the caves of Lagoa Santa, Brazil. Bull Am Mus Nat Hist 206: 414-432.

Weskler M, Percequillo A and Voss RS (2006) Ten New Genera of Oryzomyine Rodents (Cricetidae: Sigmodontinae) Am Mus Novit N°3537, 29.
