# Supplementary material for: Forest Loss and the Biodiversity Threshold: An Evaluation Considering Species Habitat Requirements and the Use of Matrix Habitats
Source: PLoS One. 2013 Dec 4;8(12):e82369. doi: 10.1371/journal.pone.0082369 (PMC3853156; doi:10.1371/journal.pone.0082369)
Supplement: Table S1 — List of small mammal species in each habitat requirement category. List of captured small mammal species, showing their classification for each of the two criteria (habitat use and geographical distribution) and the final habitat requirement classification. (DOCX) [file pone.0082369.s002.docx]

**Table S1. List of small mammal species in each habitat requirement category.** List of captured small mammal species, showing their classification for each of the two criteria (habitat use and geographical distribution) and the final habitat requirement classification.

| **Species** | | **Order** | **Geographical distribution** | | **Habitat use** |
| --- | --- | --- | --- | --- | --- |
| **Forest Specialists** |  | | |  | |
| *Didelphis aurita* | | Didelphimorpha | Centered on forested biomes | | Forest* |
| *Euryoryzomys russatus* | | Rodentia | Centered on forested biomes | | Forest |
| *Hylaeamys laticeps* | | Rodentia | Centered on forested biomes | | Forest |
| *Marmosops incanus* | | Didelphimorpha | Centered on forested biomes | | Forest |
| *Metachirus nudicaudatus* | | Didelphimorpha | Centered on forested biomes | | Forest |
| *Monodelphis americana* | | Didelphimorpha | Centered on forested biomes | | Forest |
| *Oxymycterus dasytrichus* | | Rodentia | Centered on forested biomes | | Forest |
| *Trinomys setosus* | | Rodentia | Centered on forested biomes | | Forest |
| **Habitat Generalists** |  | | |  | |
| *Akodon cursor* | | Rodentia | Encompassing also open, savanna-like biomes | | Open, anthropogenic or altered habitats, and forests |
| *Gracilinanus microtarsus* | | Didelphimorpha | Centered on forested biomes | | Open, anthropogenic or altered habitats, and forests |
| *Holochilus brasiliensis* | | Rodentia | Encompassing also open, savanna-like biomes | | Open, anthropogenic or altered habitats, and forests |
| *Marmosa murina* | | Didelphimorpha | Encompassing also open, savanna-like biomes | | Open, anthropogenic or altered habitats, and forests |
| *Micoureus demerarae* | | Didelphimorpha | Encompassing also open, savanna-like biomes | | Open, anthropogenic or altered habitats, and forests |
| *Nectomys squamipes* | | Rodentia | Encompassing also open, savanna-like biomes | | Open, anthropogenic or altered habitats, and forests |
| *Oligoryzomys nigripes* | | Rodentia | Encompassing also open, savanna-like biomes | | Open, anthropogenic or altered habitats, and forests |
| *Rhipidomys mastacalis* | | Rodentia | Centered on forested biomes | | Open, anthropogenic or altered habitats, and forests |
| **Open-area Specialists** |  | | |  | |
| *Cavia aperea* | | Rodentia | Encompassing also open, savanna-like biomes | | Open, anthropogenic or altered habitats |
| *Cerradomys vivoi* | | Rodentia | Encompassing also open, savanna-like biomes | | Open, anthropogenic or altered habitats |
| *Cryptonanus agricolai* | | Didelphimorpha | Centered on open, savanna-like biomes | | Open, anthropogenic or altered habitats |
| *Didelphis albiventris* | | Didelphimorpha | Centered on open, savanna-like biomes | | Open, anthropogenic or altered habitats |
| *Monodelphis domestica* | | Didelphimorpha | Centered on open, savanna-like biomes | | Open, anthropogenic or altered habitats |
| *Necromys lasiurus* | | Rodentia | Encompassing also open, savanna-like biomes | | Open, anthropogenic or altered habitats |
| *Pseudoryzomys simplex* | | Rodentia | Centered on open, savanna-like biomes | | Open, anthropogenic or altered habitats |
| *Rattus rattus* | | Rodentia | Exotic, introduced | | Open, anthropogenic or altered habitats |

* the species is commonly seen, but rarely captured, in anthropogenic habitats, while is very commonly captured in forests, suggesting that it depends on forest but explores open areas given its high vagility.
